# Novel homozygous variant in the *PDZD7* gene in a family with nonsyndromic sensorineural hearing loss

**DOI:** 10.1186/s12920-022-01289-7

**Published:** 2022-06-17

**Authors:** Qiang Du, Qin Sun, Xiaodong Gu, Jinchao Wang, Weitao Li, Luo Guo, Huawei Li

**Affiliations:** 1 Department of the Affiliated Eye and ENT Hospital, State Key Laboratory of Medical Neurobiology, ENT Institute and Otorhinolaryngology, Fudan University, No. 83, Fenyang Road, Shanghai, 200031 China; 2 Institutes of Biomedical Sciences, Fudan University, Shanghai, 200032 China; 3 NHC Key Laboratory of Hearing Medicine, Fudan University, Shanghai, 200031 China; 4 Shanghai Engineering Research Centre of Cochlear Implant, Shanghai, 200031 China; 5 The Institutes of Brain Science and the Collaborative Innovation Center for Brain Science, Fudan University, Shanghai, 200032 China; 6 Department of Otorhinolaryngology, Huizhou Municipal Central Hospital, Huizhou, 516002 Guangdong China

**Keywords:** Hearing loss, *PDZD7*, Variant

## Abstract

Hearing loss is the most common sensory neural disorder in humans, and according to a WHO estimation, 5.5% (466 million) of people worldwide have disabling hearing loss. In this study, a Chinese family with prelingual sensorineural hearing loss was investigated. The affected individuals showed moderately severe hearing loss at all frequencies. Using target genome enrichment and high-throughput sequencing, the homozygous variant c.2372del; p.(Ser791fs) was identified in *PDZD7*. This variant lies in exon 15 of *PDZD7* and results in a frame shift followed by an early stop codon. It is classified as pathogenic according to the ACMG/AMP guidelines and ClinGen specifications. Our study expands the pathogenic variant spectrum of *PDZD7* and strengthens the clinical importance of this gene in patients with moderately severe hearing loss.

**Supplementary Information:**

The online version contains supplementary material available at 10.1186/s12920-022-01289-7.

## Introduction

Hearing loss is the most common sensory neural disorder in humans, and according to a WHO estimation, 5.5% (466 million) of the world population has disabling hearing loss (https://www.who.int/health-topics/hearing-loss). It is estimated that more than half of neonatal sensorineural hearing loss (SNHL) is caused by genetic factors [1]. Nonsyndromic SNHL, in which no other symptoms occur, accounts for approximately 70% of hereditary SNHL. Syndromic SNHL, which is associated with other symptoms, accounts for approximately 30% of hereditary SNHL [1]. To date, 124 genes have been implicated in nonsyndromic SNHL, and more than 400 syndromic SNHLs have been identified (https://hereditaryhearingloss.org/, data updated on August 30, 2021). In the hearing system, the proteins encoded by most of these deafness genes are located in inner ear hair cells. These are polarized epithelial cells with stereocilia bundles at the top, which translate motion to neuronal signals [2].

*PDZD7* encodes a scaffold protein that is expressed in the cortex and inner ear. Pathogenic variants in *PDZD7* have been reported to cause autosomal recessive nonsyndromic SNHL [3, 4]. Moreover, *PDZD7* has been suggested to be a contributor to digenic Usher syndrome type IIC and a modifier in patients with Usher Syndrome (USH) Type IIA [5]. As the most common cause of deaf blindness, USH is divided into three subtypes (USH1, USH2, and USH3) based on the degree and onset age of hearing loss, onset age of retinitis pigmentosa and involvement of vestibular impairment. Twelve genes have been linked to USH, including 6 USH1 genes (*MYO7A*, *USH1C*, *CDH23*, *PCDH15*, *USH1G* and *CIB2*), 4 USH2 genes (*USH2A*, *ADGRV1*, *WHRN* and *PDZD7*), and 2 USH3 genes (*CLRN1* and *HARS*) [6]. Eight of these USH genes have been identified to cause both USH and nonsyndromic SNHL, namely, *MYO7A*, *CDH23*, *USH1C*, *PCDH15*, *WHRN*, *CIB2*, *USH1G* and *PDZD7*.

In this study, we present the genetic characteristics of a Chinese Han family with congenital SNHL. The affected individuals had moderately severe hearing loss at all frequencies. Using targeted genome enrichment (TGE) and high-throughput sequencing (HTS), we identified a novel homozygous frameshift variant in exon 15 of *PDZD7*. The variant results in a frame shift followed by an early stop codon and would most likely lead to nonsense-mediated mRNA decay (NMD). Our study enriches the variant spectrum of *PDZD7* and suggested that TGE and HTS are reliable tools for genetic testing of hereditary hearing loss for large genes such as *PDZD7*.

## Materials and methods

### Subjects

Participants in this study were recruited from the outpatient department of the Affiliated Eye and ENT Hospital of Fudan University, Shanghai, China. All family members were evaluated by audiological tests. Pure tone audiometry at frequencies of 125, 250, 500, 1000, 2000, 4000, and 8000 Hz was performed on family members above the age of 6. Romberg and tandem gait tests were performed to evaluate vestibular functions. Auditory brainstem response (ABR) test was performed on family members under the age of 6. High-resolution computed tomography (HRCT) scans of the temporal bone were obtained to examine inner ear malformations. Written informed consent was obtained from adult participants and parents of all minor participants involved in the study. This study was approved by the ethics committee of the Institutional Review Board of the Eye, Ear, Nose and Throat Hospital affiliated with Fudan University (Shanghai, China).

### Targeted exome sequencing

Genomic DNA was extracted from the whole blood from participants using a genomic DNA isolation kit (Qiagen, Hilden, Germany). To screen common pathogenic deafness variants in the *GJB2*, *SLC26A4*, and *MT-RNR1* genes, the patients were prescreened by PCR amplification and Sanger sequencing. A paired-end sequencing library was prepared using a library preparation kit (New England Biolabs, Ipswich, MA, catalog# E6040). A human deafness gene exon enrichment kit including 168 genes was used to capture target genome intervals (Additional file1: Table S1). High-throughput sequencing was performed using Illumina HiSeq 2000 according to the manufacturer’s instructions (Illumina, Inc., San Diego, CA).

### Bioinformatics and validation of the variants

Sequencing reads were generated by the Illumina CASAVA v1.8 pipeline and aligned to the human reference genome (hg19) using the Burrows–Wheeler Aligner (BWA) program. Variants were called using the GATK package v4.1.8.1. All variants were annotated and characterized using ANNOVAR software. To identify pathogenic variants, we filtered out the following: (1) low-quality variants (depth < 10, or genotype quality < 30); (2) variants in the noncoding regions, except for those that might disrupt splicing; (3) synonymous variants in the coding region; (4) variants with minor allele frequency (MAF) > 0.001 in several databases (1000 Genome Project, gnomad v2.1.1 and in-house database); and (5) variants labeled as “benign” in the ClinVar database. The deleterious effect of variants was predicted by SIFT scores, REVEL, and CADD scores. To validate the variants, Sanger sequencing of *PDZD7* exon 15 was performed on genomic DNA from all family members and 96 normal hearing controls. PCR and sequencing primers were designed by Primer3 online software. Sanger sequencing was performed on a 3730XL sequencer (Applied Biosystems) according to the manufacturer’s instructions.

## Results

### Family and clinical presentations

**Fig. 1:**
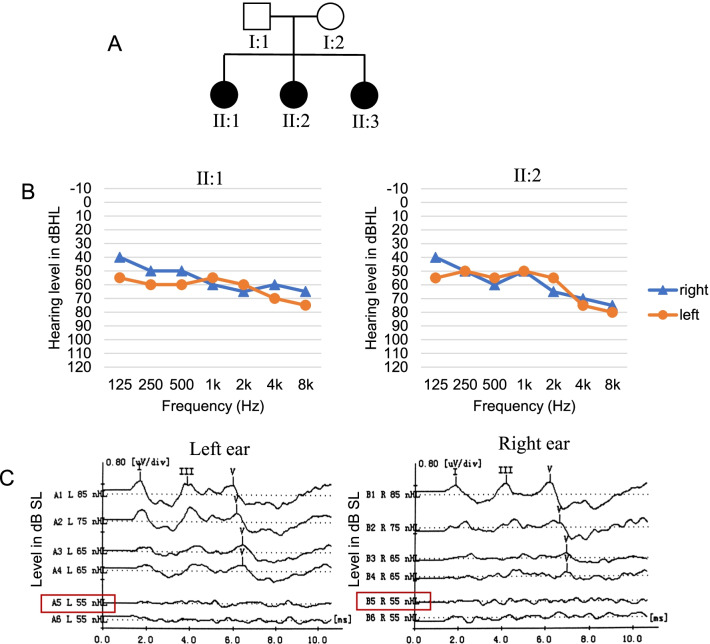
Pedigree and audiograms of the hearing loss family. **A** Pedigree of the family. Darkened symbols denote affected individuals. **B** Audiograms of the two affected Siblings (II:1 and II:2) in the family. **C** ABR results of II:3

Family D27 is a nonconsanguineous Chinese family that includes three affected siblings and two normal hearing parents (Fig. 1A). This family underwent auditory tests, and the family history was obtained. The affected siblings were 9, 7 and 2 years old during the examination. Audiograms of the two older patients showed bilateral moderate to severe HL with a slightly downward slope (Fig. 1B). The ABR test of II:3 revealed bilateral HL with a threshold of approximately 65 dB (Fig. 1C). Although newborn hearing screenings were not performed, the parents recalled no responses to subtle sounds at 1–2 years of age, suggesting a congenital phenotype. Vestibular functional tests of the two older patients revealed no abnormalities. HRCT scans of II:1 and II:2 revealed no inner ear malformations. We also performed pure tone audiometry on the parents and detected no hearing loss features. Otoscopy and full physical examination with special attention to renal and ophthalmological evaluations revealed no additional abnormalities.

### Targeted high-throughput sequencing

Targeted high-throughput sequencing of all exons and exon–intron boundaries for 168 deafness genes was performed on the proband II:1. Sequencing yielded 2.8 million 100 bp paired-end reads. After adaptor trimming and low-quality read filtering, paired-end fastq files were aligned to the human genome (hg19). A mean depth of 118.1 for the targeted exons was achieved, and 97.8% of the targeted genome intervals were covered by at least 10 sequencing reads. After filtering against MAFs from various databases (1000 Genome Project, gnomad v2.1.1 and in-house database), we focused on variants in the coding region and intronic variants that might affect splicing. Based on the assumption of an autosomal recessive mode of inheritance, we focused on genes with homozygous or compound heterozygous variants. A homozygous variant, c.2372del; p.(Ser791Phefs*17); p.(Ser791fs) (NM_001195263.2), was identified in exon 15 of *PDZD7*. This variant was not present in any of the reference databases.

### Genetic analysis of the *PDZD7* variant

**Fig. 2:**
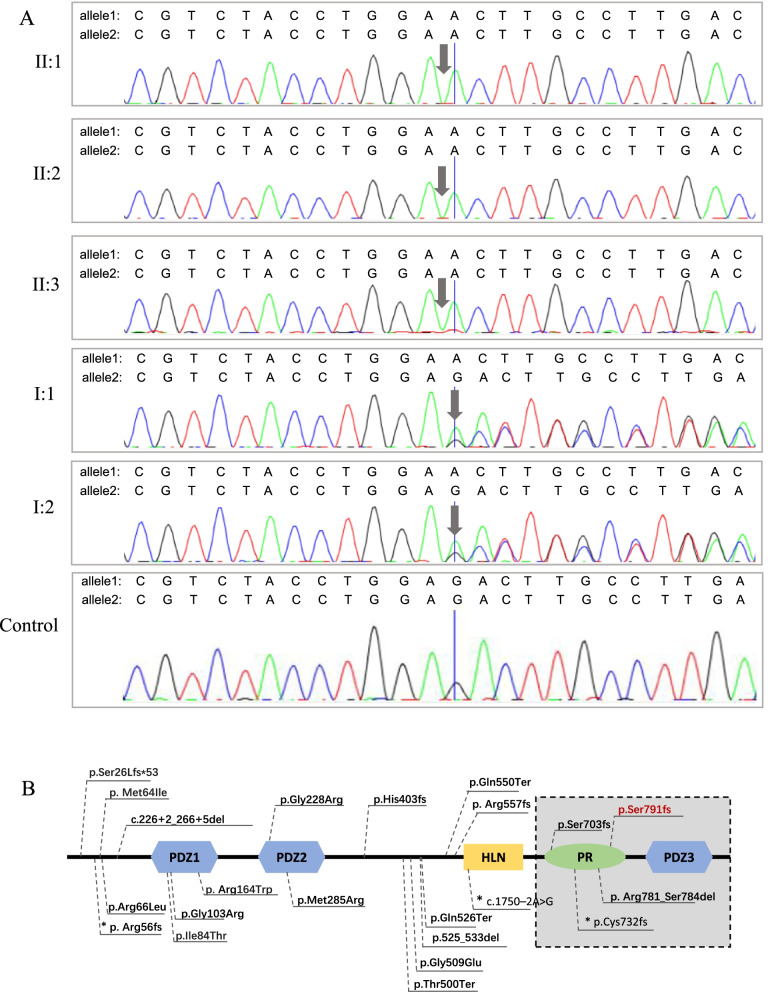
Sanger sequencing of the pathogenic variant. **A** Sanger sequencing chromatograms showing the c.2372del; p.(Ser791fs) variant in the homozygous state in affected individuals II:1, II:2, and II:3 compared with the heterozygous sequence in individuals I:1 and I:2 and an ethnic-matched normal hearing control. Arrows indicate the location of the variant. The reverse strand was sequenced. **B** Schematic representation of the PDZD7 protein. The novel variant c.2372del; p.(Ser791fs) identified in this study is red. The gray rectangle indicates the domains of the PDZD7 protein that are unique to the long isoform. Variants identified as Usher syndrome modifiers are denoted with *

Sanger sequencing of exon 15 of *PDZD7* was performed for all family members (Fig. 2A). Variant interpretation was performed according to the ACMG/AMP guidelines and ClinGen specifications [7–10]. Loss of function is a known mechanism of *PDZD7*-induced HL [11]. The frameshift variant p.(Ser791fs) is followed by an early stop codon (Fig. 2A). The premature termination codon lies in the middle of the antepenultimate exon of *PDZD7* and therefore would most likely cause NMD (PVS1) [12, 13]. The three affected siblings were homozygous for the *PDZD7* variant, while their normal hearing parents were both heterozygous, which suggests that the *PDZD7* variant cosegregated with HL in this family (PP1). Moreover, the variant was absent in the 1000 Genome Project, gnomad v2.1.1 and in-house databases, and Sanger sequencing of 96 ethnically matched normal hearing controls did not detect the variant (PM2). Therefore, the c.2372del; p.(Ser791fs) (NM_001195263.2) variant is classified as pathogenic according to the ACMG/AMP guidelines and ClinGen specifications, with the applied criteria of PVS1, PM2 and PP1 [8, 9].

## Discussion

*PDZD7* is located on chromosome 10q24.31 and was originally identified as an autosomal recessive nonsyndromic SNHL gene [4]. Subsequently, heterozygous variants in *PDZD7* were identified as a modifier of retinal disease and a contributor to digenic Usher syndrome [5]. In Usher syndrome patients with biallelic *USH2A* variants, another heterozygous *PDZD7* variant causes earlier-onset and more severe retinal disease [5]. Moreover, heterozygous variants in both *PDZD7* and *ADGRV1* induce Usher Syndrome Type IIC [5]. In this study, using HTS, we identified a novel pathogenic *PDZD7* variant (c.2372del; p.(Ser791fs)) in a Chinese Han family with congenital nonsyndromic SNHL. The variant is located in the middle of exon 15, which is the antepenultimate exon. According to current consensus, this premature termination codon will lead to NMD, and no protein will be produced [12, 13]. Furthermore, no other variants in the Usher syndrome genes were found in the proband. The affected siblings in the present study were 9, 7 and 2 years of age during examinations. Ophthalmological tests revealed no retinal abnormalities. Newborn hearing screenings were not performed, but the parents recalled a reduced response to small sounds and mispronunciations for the two older sisters, which indicated congenital or prelingual onset.

The PDZD7 protein is a paralog of harmonin (*USH1C*) and whirlin (*WHRN*), sharing 35% and 55% similarity with harmonin and whirlin, respectively [11]. It contains three PDZ domains, a harmonin-N-like domain (HNL) and a proline-rich (PR) region. *PDZD7* is expressed in inner ear hair cells and forms an Usher quaternary protein complex with USH2A, ADGRV1 and WHRN. This complex is essential for the development and organization of the ankle-link complex, which localizes at the ankle region of hair cell stereocilia [11, 14]. The first two PDZ domains of PDZD7 mediate interaction with the other Usher quaternary protein complex components USH2A, ADGRV1 and WHRN, whereas the third PDZ domain is only involved in the interaction with WHRN [14].

To date, 22 pathogenic *PDZD7* variants have been reported in the literature, 3 of which are modifier variants of USH2A (Fig. 2B; Table 1) [3, 5, 15, 16, 18–22]. We summarized the features of these variants, including the phenotypes of the patients and characteristics of the variants, in Table 1. These variants are spread along the gene without any hotspots (Table 1). Patients with biallelic *PDZD7* variants showed prelingual moderate to severe hearing loss with a downsloping audiogram. Hearing loss may be progressive in some patients. Three USH modifier variants have been identified thus far. These include two frameshift variants and one splicing variant (p.Arg56fs, c.1750-2A > G and p.Cys732fs), all of which are predicted to induce NMD. These USH modifier variants show no difference in pathogenic mechanisms compared with other monogenic *PDZD7* variants. Moreover, p.Arg56fs was also found in nonsyndromic patients. Four alternative splicing isoforms of *PDZD7* have been detected; they encode either a full-length protein or short isoforms mainly containing the first two PDZ domains [4]. The variant identified in this study localizes to exon 15 of *PDZD7*, which is unique to the long isoform (Fig. 2B). Including our research, 3 pathogenic variants have been discovered in domains unique to the long isoform (Fig. 2B). Patients harboring each of these 3 variants (p.Ser703fs, p.Arg781_Ser784del and p.(Ser791fs)) showed the same characteristic auditory phenotype of bilateral moderately severe hearing loss at all frequencies with gentle downward sloping as patients with variants in other parts of the gene [15, 16]. Another variant (p.Cys732fs), cosegregating with biallelic USH2A variants in a patient with Usher syndrome, unique to the long isoform, was identified as a Usher syndrome modifier [5]. A mouse model lacking exons 2–5 of *Pdzd7*, which disrupts all isoforms, and a mouse model lacking exon 14, which only disrupts the long isoform, both manifest stereocilia disorganization and MET deficits, leading to a similar hearing loss phenotype. Moreover, in mice lacking exon 14 of the *Pdzd7* gene, the short isoforms were not detected in the inner ear at the protein level. These findings suggest that the *PDZD7* long isoform is indispensable for hair cell function. However, the *PDZD7* short isoform may not localize in the stereocilia and therefore make no contribution to stereocilia function.

**Table 1:** Summary of all reported *PDZD7* variants to date

ID	Origin	Disease	Genotype	Mutation type	HGVS.cDNA	HGVS.protein	Location	Domain	Age of onset	Auditory threshold	Auditory profile	References
1	China	NS-SNHL	Homo	Frameshift	c.2372del	p.Ser791fs	Exon 15	PR	Congenital	Moderate to severe	Down-sloping	This study
2	China	NS-SNHL	Comp Het	Missense and Nonsense	c.192G > A and c.1648C > T	p.Met64Ile and p.Gln550Ter	Exon 2	–*	Prelingual	Mild to moderate	Down-sloping	[16]
			Comp Het	In-frame deletion	c.2341_2352del	p.Arg781_Ser784del	Exon 15	PR				
3	Germany	NS-SNHL	Comp Het	Nonsense	c.1648C > T	p.Gln550Ter	Exon 10	–	Prelingual	Moderate to severe	Down-sloping	[17]
			Comp Het	Frameshift	c.2107del	p.Ser703fs	Exon 15	PR				
4	China	NS-SNHL	Comp Het	Frameshift	c.166_167insC	p.Arg56fs	Exon 2	–	Prelingual	Moderate to severe	Down-sloping	[18]
			Comp Het	Frameshift	c.1207del	p.His403fs	Exon 8	–				
5	China	NS-SNHL	Homo	Missense	c.197G > T	p.Arg66Leu	Exon 2	–	Prelingual	Moderate to severe	Down-sloping	[18]
6	Iranian	NS-SNHL	Homo	Missense	c.307G > C	p.Gly103Arg	Exon 3	PDZ1	Prelingual	Moderate to severe	Down-sloping	[3]
7	Iranian	NS-SNHL	Homo	Missense	c.682G > A	p.Gly228Arg	Exon 5	PDZ2	Prelingual	Severe	Flat	[3]
8	Iranian	NS-SNHL	Comp Het	Missense	c.854 T > G	p.Met285Arg	Exon 6	PDZ2	Prelingual	Moderate to severe	Down-sloping	[3]
			Comp Het	Nonsense	c.1500C > A	p.Thr500Ter	Exon 9	–				
9	Iranian	NS-SNHL	Homo	Nonsense	c.1576C > T	p.Gln526Ter	Exon 9	–	Prelingual	Severe	Flat	[3]
10	South Korea	NS-SNHL	Homo	Missense	c.490C > T	p.Arg164Trp	Exon 4	PDZ1	Prelingual	Moderate to severe	NA	[19]
11	South Korea	NS-SNHL	Comp Het	Missense	c.490C > T	p.Arg164Trp	Exon 4	PDZ1	Prelingual	Severe	NA	[19]
			Comp Het	Frameshift	c.1669del	p.Arg557fs	Exon 11	HNL				
12	South Korea	NS-SNHL	Comp Het	Missense	c.490C > T	p.Arg164Trp	Exon 4	PDZ1	prelingual	Moderate to severe	NA	[19]
			Comp Het	Missense	c.1526G > A	p.Gly509Glu	Exon 10	–				
13	Pakistani	NS-SNHL	Homo	Splicing	c.226 + 2_226 + 5del	–	Intron 2	–	Congenital	Moderate	NA	[20]
14	Iranian	NS-SNHL	Homo	Missense	c.251 T > C	p.Ile84Thr	Exon 3	PDZ1	NA	Severe	Down-sloping	[21]
15	China	NS-SNHL	Comp Het	In-frame deletion	c.1574_1597del	p.Asp525_Leu533del	Exon 9	–	congenital	Moderate	NA	[22]
		NS-SNHL	Comp Het	Missense	c.490C > T	p.Arg164Trp	Exon 4	PDZ1				
16	South Korea	NS-SNHL	Het/Digenic with ADGRV1	Frameshift	c.76_77del	p.Ser26fs	Exon 2	–	prelingual	Mild to moderate	Down-sloping	[23]
17	France	Usher syndrome type 2	Het/Usher modifier/co-segregate with biallelic USH2A variants	Frameshift	c.166_167insC	p.Arg56fs	Exon 2	–	prelingual	Moderate	NA	[5]
18	Germany	Usher syndrome type 2	Het/Usher modifier/co-segregate with biallelic USH2A variants	Splicing	c.1750-2A > G	–	Intron 11	–	Prelingual	Moderate	NA	[5]
19	Germany	Usher syndrome type 2	Het/Digenic with ADGRV1	Frameshift	c.2194_2203del	p.Cys732fs	Exon 15	PR	Diagnosed at age 5	Moderate to severe	NA	[5]

*Homo* homozygosity, *Het* heterozygosity, *Comp Het* compound heterozygosity, *NA* not available, *PR* proline-rich domain, *HNL* harmonin-N-like domain, *PDZ*, PDZ domain

*“–” denotes that the variant lies in the protein where no domains were identified

Hereditary hearing loss is a genetically and phenotypically heterozygous disorder. To date, 124 genes have been identified for nonsyndromic SNHL, and 46 genes have been identified for the nine most common syndromic HLs (https://hereditaryhearingloss.org/, data updated on August 30, 2021). The phenotypes of the patients vary by audiogram, age of onset, progression, vestibular complications, inner ear malformations, retinal complications, etc. [23]. These heterogeneities hindered the genetic diagnosis of hearing loss and call for a more comprehensive variant screening strategy that takes phenotype-genotype correlations into consideration. Since most cases of autosomal recessive nonsyndromic SNHL is characterized by prelingual severe to profound HL, the relatively rare moderately severe audiogram at all frequencies may serve as a reminder for potential causative *PDZD7* variants.

We report a novel pathogenic frameshift variant on *PDZD7* in a Chinese family with moderately severe HL. This variant lies in exon 15 and is unique to the long isoform of the PDZD7 protein. Our study extends the variant spectrum of the *PDZD7* gene in the Chinese population. The relatively uncommon moderately severe audiogram with a slightly downward slope is characteristic of *PDZD7* patients. The identification of a novel pathogenic *PDZD7* variant may be valuable for genetic consultation and functional research.

## Supplementary Information

**Additional file1.** **Supplementary Table S1**: Table of genes for the hearing loss sequencing panel.

## Data Availability

The datasets used and/or analyzed during the current study are available from the corresponding author upon reasonable request.

## References

[1] Smith RJ, Bale JF, White KR (2005). Sensorineural hearing loss in children. Lancet.

[2] Dror AA, Avraham KB (2010). Hearing impairment: a panoply of genes and functions. Neuron.

[3] Booth KT, Azaiez H, Kahrizi K, Simpson AC, Tollefson WTA, Sloan CM, Meyer NC, Babanejad M, Ardalani F, Arzhangi S (2015). PDZD7and hearing loss: more than just a modifier. Am J Med Genet A.

[4] Schneider E, Märker T, Daser A, Frey-Mahn G, Beyer V, Farcas R, Schneider-Rätzke B, Kohlschmidt N, Grossmann B, Bauss K (2009). Homozygous disruption of PDZD7 by reciprocal translocation in a consanguineous family: a new member of the Usher syndrome protein interactome causing congenital hearing impairment. Hum Mol Genet.

[5] Ebermann I, Phillips JB, Liebau MC, Koenekoop RK, Schermer B, Lopez I, Schäfer E, Roux A-F, Dafinger C, Bernd A (2010). PDZD7 is a modifier of retinal disease and a contributor to digenic Usher syndrome. J Clin Investig.

[6] Toms M, Pagarkar W, Moosajee M (2020). Usher syndrome: clinical features, molecular genetics and advancing therapeutics. Ther Adv Ophthalmol.

[7] Zhang J, Yao Y, He H, Shen J (2020). Clinical interpretation of sequence variants. Curr Protoc Hum Genet.

[8] Richards S, Aziz N, Bale S, Bick D, Das S, Gastier-Foster J, Grody WW, Hegde M, Lyon E, Spector E (2015). Standards and guidelines for the interpretation of sequence variants: a joint consensus recommendation of the American College of Medical Genetics and Genomics and the Association for Molecular Pathology. Genet Med.

[9] Oza AM, DiStefano MT, Hemphill SE, Cushman BJ, Grant AR, Siegert RK, Shen J, Chapin A, Boczek NJ, Schimmenti LA (2018). Expert specification of the ACMG/AMP variant interpretation guidelines for genetic hearing loss. Hum Mutat.

[10] Peng J, Xiang J, Jin X, Meng J, Song N, Chen L, Abou Tayoun A, Peng Z (2021). VIP-HL: Semi-automated ACMG/AMP variant interpretation platform for genetic hearing loss. Hum Mutat.

[11] Zou J, Zheng T, Ren C, Askew C, Liu X-P, Pan B, Holt JR, Wang Y, Yang J (2014). Deletion of PDZD7 disrupts the Usher syndrome type 2 protein complex in cochlear hair cells and causes hearing loss in mice. Hum Mol Genet.

[12] Abou Tayoun AN, Pesaran T, DiStefano MT, Oza A, Rehm HL, Biesecker LG, Harrison SM (2018). ClinGen Sequence Variant Interpretation Working G: recommendations for interpreting the loss of function PVS1 ACMG/AMP variant criterion. Hum Mutat.

[13] Supek F, Lehner B, Lindeboom RGH (2021). To NMD or Not To NMD: nonsense-mediated mRNA decay in cancer and other genetic diseases. Trends Genet.

[14] Chen Q, Zou J, Shen Z, Zhang W, Yang J (2014). Whirlin and PDZ domain-containing 7 (PDZD7) proteins are both required to form the quaternary protein complex associated with usher syndrome type 2. J Biol Chem.

[15] Luo H, Hassan RN, Yan J, Xie J, Du P, Hu Q, Zhu Y, Jiang W (2019). Novel recessive PDZD7 biallelic mutations associated with hereditary hearing loss in a Chinese pedigree. Gene.

[16] Vona B, Lechno S, Hofrichter MA, Hopf S, Laig AK, Haaf T, Keilmann A, Zechner U, Bartsch O (2016). Confirmation of PDZD7 as a nonsyndromic hearing loss gene. Ear Hear.

[17] Guan J, Wang H, Lan L, Wang L, Yang J, Xie L, Yin Z, Xiong W, Zhao L, Wang D (2018). Novel recessive PDZD7 biallelic mutations in two Chinese families with non-syndromic hearing loss. Am J Med Genet A.

[18] Lee SY, Han JH, Kim BJ, Oh SH, Lee S, Oh DY, Choi BY (2019). Identification of a potential founder effect of a novel PDZD7 Variant involved in moderate-to-severe sensorineural hearing loss in Koreans. Int J Mol Sci.

[19] Le Quesne SP, James C, Ocaka L, Tekman M, Grunewald S, Clement E, Stanescu HC, Kleta R, Morrogh D, Calder A (2017). An example of the utility of genomic analysis for fast and accurate clinical diagnosis of complex rare phenotypes. Orphanet J Rare Dis.

[20] Fahimi H, Behroozi S, Noavar S, Parvini F (2021). A novel recessive PDZD7 bi-allelic mutation in an Iranian family with non-syndromic hearing loss. BMC Med Genom.

[21] Wu D, Huang W, Xu Z, Li S, Zhang J, Chen X, Tang Y, Qiu J, Wang Z, Duan X (2020). Clinical and genetic study of 12 Chinese Han families with nonsyndromic deafness. Mol Genet Genom Med.

[22] Kim NK, Kim AR, Park KT, Kim SY, Kim MY, Nam JY, Woo SJ, Oh SH, Park WY, Choi BY (2015). Whole-exome sequencing reveals diverse modes of inheritance in sporadic mild to moderate sensorineural hearing loss in a pediatric population. Genet Med.

[23] Shearer AE, Hildebrand MS, Sloan CM, Smith RJ (2011). Deafness in the genomics era. Hear Res.

