# Supplementary material for: Novel homozygous variant in the PDZD7 gene in a family with nonsyndromic sensorineural hearing loss
Source: BMC Med Genomics. 2022 Jun 17;15:135. doi: 10.1186/s12920-022-01289-7 (PMC9204979; doi:10.1186/s12920-022-01289-7)
Supplement: Supplementary file 1 — Additional file1. Supplementary Table S1: Table of genes for the hearing loss sequencing panel. [file 12920_2022_1289_MOESM1_ESM.docx]

Supplementary table 1

| ACTG1 | DIAPH3 | HARS2 | MYO6 | RDX | TPRN |
| --- | --- | --- | --- | --- | --- |
| ALX3 | DSPP | HGF | MYO7A | RPGR | TRIOBP |
| BSND | ECM1 | HMX1 | NDP | SALL1 | TRMU |
| CABP2 | EDN3 | HOXA2 | NDRG1 | SALL4 | TSPEAR |
| CCDC50 | EDNRB | HSD17B4 | NEFL | SEC23A | TYR |
| CDH23 | ELMOD3 | IL13 | NELL2 | SEMA3E | USH1C |
| CEACAM16 | ESPN | ILDR1 | NF2 | SERPINB6 | USH1G |
| CHD7 | ESRRB | KARS | OPA1 | SIX1 | USH2A |
| CIB2 | EYA1 | KCNE1 | OTOA | SIX5 | WFS1 |
| CLDN14 | EYA4 | KCNJ10 | OTOF | SLC17A8 | MT-RNR1 |
| CLPP | FGF3 | KCNQ1 | OTOG | SLC19A2 | MT-TL1 |
| CLRN1 | FGF8 | KCNQ4 | OTOGL | SLC26A4 | MT-CO1 |
| COCH | FGFR1 | KITLG | P2RX2 | SLC26A5 | MT-TS1 |
| COL11A1 | FGFR3 | KRT9 | PABPN1 | SMAD4 | MT-TK |
| COL11A2 | FLNA | LAMA3 | PAX3 | SMPX | MT-TE |
| COL1A1 | FOXI1 | LARS2 | PCDH15 | SNAI2 | miR-96 |
| COL1A2 | FREM1 | LHFPL5 | PCDH9 | SOX10 | miR-182 |
| COL2A1 | FXN | LOXHD1 | PDZD7 | STRC | miR-183 |
| COL4A3 | GATA3 | LRTOMT | PMP22 | TBC1D24 |  |
| COL4A4 | GIPC3 | MARVELD2 | PNPT1 | TCIRG1 |  |
| COL4A5 | GJB1 | MIR96 | POLR1C | TCOF1 |  |
| COL4A6 | GJB2 | MITF | POLR1D | TECTA |  |
| COL9A1 | GJB3 | MPZ | POU3F4 | TIMM8A |  |
| COL9A2 | GJB6 | MSRB3 | POU4F3 | TJP2 |  |
| CRYM | GLYAT | MYH14 | PROK2 | TMC1 |  |
| DFNA5 | GPR98 | MYH9 | PROKR2 | TMEM126A |  |
| DFNB31 | GPSM2 | MYO15A | PRPS1 | TMIE |  |
| DFNB59 | GRHL2 | MYO1A | PTPN11 | TMPRSS3 |  |
| DIABLO | GRXCR1 | MYO1E | PTPRQ | TMPRSS4 |  |
| DIAPH1 | HARS | MYO3A | PTPRR | TNC |  |

Table legends:

Supplementary table 1: Table of genes for the hearing loss sequencing panel.
